# Induced Mutagenesis in *UGT74S1* Gene Leads to Stable New Flax Lines with Altered Secoisolariciresinol Diglucoside (SDG) Profiles

**DOI:** 10.3389/fpls.2017.01638

**Published:** 2017-09-21

**Authors:** Bourlaye Fofana, Kaushik Ghose, Ashok Somalraju, Jason McCallum, David Main, Michael K. Deyholos, Gordon G. Rowland, Sylvie Cloutier

**Affiliations:** ^1^Charlottetown Research and Development Centre, Agriculture and Agri-Food Canada Charlottetown, PE, Canada; ^2^Department of Biology, University of British Columbia Kelowna, BC, Canada; ^3^Department of Plant Science, Crop Development Centre, University of Saskatchewan Saskatoon, SK, Canada; ^4^Ottawa Research and Development Centre, Agriculture and Agri-Food Canada Ottawa, ON, Canada

**Keywords:** flax, lignan, *UGT74S1*, EMS, SNPs, secoisolariciresinol, reverse genetics, forward genetics

## Abstract

Flax secoisolariciresinol (SECO) diglucoside (SDG) lignan is an emerging natural product purported to prevent chronic diseases in humans. SECO, the aglycone form of SDG, has shown higher intestinal cell absorption but it is not accumulated naturally *in planta*. Recently, we have identified and characterized a UDP-glucosyltransferase gene, *UGT74S1*, that glucosylates SECO into its monoglucoside (SMG) and SDG forms when expressed in yeast. However, whether this gene is unique in controlling SECO glucosylation into SDG *in planta* is unclear. Here, we report on the use of *UGT74S1* in reverse and forward genetics to characterize an ethyl methane sulfonate (EMS) mutagenized flax population from cultivar CDC Bethune and consisting of 1996 M2 families. EMS mutagenesis generated 73 SNP variants causing 79 mutational events in the *UGT74S1* exonic regions of 93 M2 families. The mutation frequency in the exonic regions was determined to be one per 28 Kb. Of these mutations, 13 homozygous missense mutations and two homozygous nonsense mutations were observed and all were transmitted into the M3 and M4 generations. Forward genetics screening of the population showed homozygous nonsense mutants completely lacking SDG biosynthesis while the production of SMG was observed only in a subset of the M4 lines. Heterozygous or homozygous M4 missense mutants displayed a wide range of SDG levels, some being greater than those of CDC Bethune. No additional deleterious mutations were detected in these mutant lines using a panel of 10 other genes potentially involved in the lignan biosynthesis. This study provides further evidence that *UGT74S1* is unique in controlling SDG formation from SECO and this is the first report of non-transgenic flax germplasm with simultaneous knockout of SDG and presence of SMG *in planta*.

## Introduction

Flax lines with high linolenic acid (Duguid et al., 2014), low linolenic acid (Green and Marshall, 1984; Rowland, 1991; Dribnenki et al., 1996), and low cyanogenic glucosides (Duguid, personal communications) are some of the major achievements in linseed genetics and breeding. Whereas, the first two attributes are associated with fatty acid content, the last one refers to flax seed content in linustatin and neolinustatin, two cyanogenic glucosides that are toxic to mammals (EFSA, 2007). The nutritional benefits of flaxseed linolenic acid in human and animal health are now well established, especially for the positive roles it plays in reducing incidence of cardiovascular diseases (Simopoulos, 2008). Flaxseed is also the richest source of lignans (Thompson et al., 2006; Pan et al., 2009; Touré and Xueming, 2010) for which a wide variety of purported health benefits have been reported (Webb and McCullough, 2005; Adolphe et al., 2010; Buck et al., 2011; Wang et al., 2015). *In planta*, flax lignans are usually found as secoisolariciresinol diglucosides (SDGs) ester-linked within an oligomeric matrix called the lignan macromolecule (Struijs et al., 2009; Kosińska et al., 2011). Its monomeric aglycone (SECO) and intermediate monoglucoside (SMG) forms do not naturally accumulate *in planta*.

Lignan metabolism in mammals has been the focus of many detailed studies (During et al., 2012; Udani et al., 2013; Setchell et al., 2014; Mukker et al., 2015). After consumption, the lignan macromolecule complex is hydrolyzed, SDG is deglucosylated into SECO and absorbed in the gut (Clavel et al., 2007). Non-absorbed SECO (50–72% of ingested SECO) is subsequently metabolized into the enterolignans, enterolactone (ENL), and enterodiol (END) mainly in the colon by the intestinal microflora (Clavel et al., 2006, 2007; Landete et al., 2015). Enterolignans have been reported to elicit modest estrogen-like activity by binding estrogen receptors (ERs), ERα or ERβ (Satake et al., 2015). Nonetheless, low concentrations of intact lignans have been detected in the sera of mammals fed lignan-rich diets, suggesting that non-metabolized lignans are taken up by the mammalian digestive system and manifest ER-independent activities *in vivo* and *in vitro* (During et al., 2012; Satake et al., 2015). As a β-glucoside however, SDG metabolism may vary between individuals due to differences in their gut microflora (Rowland et al., 2000; Clavel et al., 2005; Landete et al., 2015), thereby affecting its bioavailability in some individuals (Possemiers et al., 2007) similar to what has already been shown with isoflavone diazein, which is metabolized only by one third of humans into its bioactive S-equol form (Setchell and Cole, 2006; Landete et al., 2015). Recently, During et al. (2012) reported a linear increase in the uptake of lignan aglycone forms, including pinolariciresinol (PINO), SECO, and ENL, by human intestinal Caco-2 cells by simple diffusion or low affinity transporter. Less than 0.1% SDG absorption was observed, compared to 2% for SECO and PINO, and 7% for ENL, evidencing the effect of glucosylation on absorption and bioavailability. However, no SMG of flax lignan has been available so far for such testing. Due to its lower molecular weight of 524.6 g/mol and the lack of a second bulky and polar glucoside moiety, it is logical to conceive that SMG uptake by Caco-2 cells through diffusion would exceed that of SDG which has a molecular weight of 686.7 g/mol. Currently, SECO and SMG are only obtained after acid hydrolysis of SDG (Li et al., 2008), a process that is not environmentally friendly. Because SDG deglucosylation is a pre-requisite to any absorption or conversion into END and ENL *in vivo*, production of seeds with altered SDG glucosylation *in vivo* is of interest for designing a highly bioavailable functional flaxseed food. It would be advantageous to create flax lines with reduced lignan glucosylation in the forms of SECO or SMG *in planta*.

**Figure 1:**
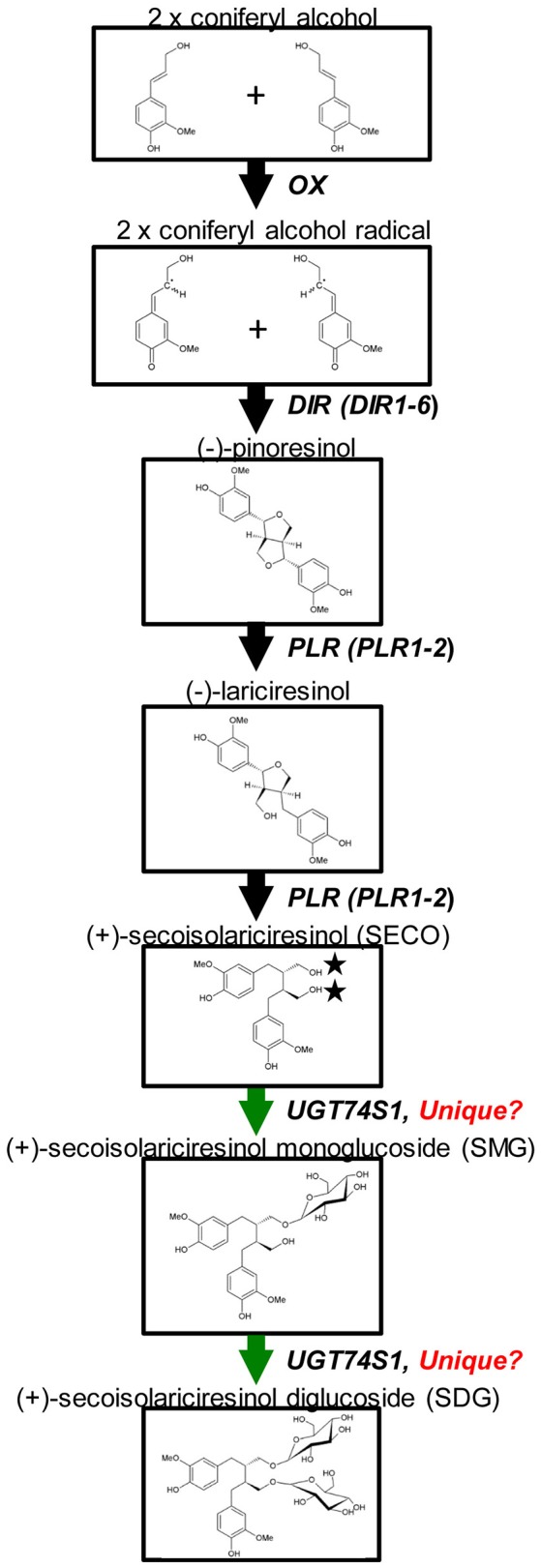
Current knowledge of flax lignan biosynthetic pathways. Genes with known functions are shown (adapted from Kim et al., 2009) with the permission of Dr. Honoo Satake and PCP editorial office and from Ghose et al. (2014) with the permission of BMC Plant Biology editorial office.

Glycosylation is a biological process that leads to chemical complexity and diversity of plant natural products (Osmani et al., 2009; Wang and Hou, 2009). This synthesis modification is catalyzed by enzymes of the glycosyltransferases (GT) superfamily in which family 1 GT, refers to uridine glycosyl transferases (UGTs) (Caputi et al., 2012) that are characterized by a PSPG box consisting of a 44 amino acid consensus signature motif (Gachon et al., 2005). UGTs transfer UDP-activated sugar moieties to specific acceptor molecules (Witte et al., 2009) and their glycosylation activities on acceptors can modulate their pharmacological activity (Osmani et al., 2009). Recently, UGT74S1 (JX011632), a family 1 GT, was identified in flax and characterized to sequentially glucosylate SECO into SMG and SDG (Ghose et al., 2014; Figure 1). *UGT74S1* has also been reported to be a single-copy gene and the key player in controlling SDG formation in flax (Fofana et al., 2017). Furthermore, Trp^355^ and His^352^ were found to be critical for the UGT74S1 glucosylation activity toward SECO whereas Gln^337^ and Ser^357^ appeared essential for SMG conversion into SDG *in vitro* (Ghose et al., 2015). Until now however, no study has assessed the direct effect of *UGT74S1* mutations *in planta*. Moreover, despite growing interests in flax lignans, breeding for SDG *per se* has so far received little attention (Pavelek et al., 2015), with most studies being limited to observed variations among flax varieties and accessions within collections (Saastamoinen et al., 2013). So far, no flax lines with altered lignan glucosylation have been reported.

Flaxseed is a health-functional crop and the commercial production of genetically modified flax remains an impediment to conquering such markets (Fofana et al., 2010). Mutation breeding is a well-established method in flax improvement and has led to the development of cultivars with reduced linolenic acid (Green and Marshall, 1984; Rowland et al., 1995; Ntiamoah and Rowland, 1997). Targeting Induced Local Lesions in Genomes (TILLING) is a reverse genetic tool used in functional genomics and crop improvement (McCallum et al., 2000; Sikora et al., 2011). Traditional mutagenesis, including radiation or chemical mutagenesis, is followed by the detection of mutations in the causal gene(s) responsible for the phenotype(s) of interest (McCallum et al., 2000; Slade and Knauf, 2005; Uchida et al., 2011; Chantreau et al., 2013). Recently, for example, Chantreau et al. (2013) developed a flax EMS mutagenized population and characterized the roles of coumarate-3-hydroxylase (C3H) and cinnamyl alcohol dehydrogenase (CAD) in fiber biosynthesis in flax stems by TILLING via forward and reverse genetics. The chemical mutagen, ethyl methane sulfonate (EMS), generates a wide range of mutant alleles, primarily point mutations (Comai and Henikoff, 2006) and, current advances in next generation sequencing have greatly improved the efficiency of induced-mutation detection (Galindo-González et al., 2015). With the release of the flax genome sequence (Wang et al., 2012), the DNA sequence of almost all flax genes is known. However, the gene functions and their link to phenotypic traits remain largely incomplete or completely unknown.

The present study was undertaken to (1) characterize an EMS mutagenized flaxseed population through the identification of lines carrying mutations in *UGT74S1* and displaying altered lignan glucosylation phenotypes, and (2) determine the extent of the role played by UGT74S1 in SDG formation through SECO glucosylation. Using a next generation sequencing platform for the amplicon re-sequencing of the *UGT74S1* gene in an EMS mutagenized flax population, we identified stable mutant lines with altered SDG lignan profile and demonstrated that the loss-of-function of SECO glucosylation into SDG was attributable solely to UGT74S1.

## Materials and methods

### Plant materials

A total of 1996 M2 EMS mutagenized flax families were obtained from Dr. Gordon G. Rowland (Crop Development Centre, University of Saskatchewan, Saskatoon, SK) who multiplied them before distribution. These M2 families were originally developed by Dr. Michael K. Deyholos' laboratory (University of Alberta, Edmonton, AB) as part of the Genome Canada's Total Utilization Flax GENomics (TUFGEN) project. To generate these lines, seeds of flax (*Linum usitatissimum* L.) cultivar CDC Bethune (Rowland et al., 2002) were treated with 0.5% (v/v) EMS for 4 h, planted and grown to seed set. M2 seeds from M1 plants were harvested separately as previously described (Galindo-González et al., 2015). Five seeds from each of the 1996 M2 families were hand sown in 30 cm rows in 2011 at the Harrington Research Farm of Agriculture and Agri-Food Canada (Charlottetown, PEI) along with 10 rows of untreated CDC Bethune. Individual EMS plants within a row are referred to as lines and, all plants within a row constitute a family. Leaves of one line from each M2 family and 20 CDC Bethune plants were collected, frozen in liquid nitrogen and stored at −80°C for DNA extraction and reverse genetic analysis. All M2 plants were destroyed before flowering to avoid any potential cross pollination and seed production in the field. Re-sampling from the original M2 seed stock was also performed, whenever necessary, by planting in greenhouse to generate subsequent mutant generations.

### Reverse genetics of *UGT74S1*

#### Genomic DNA extraction and targeted *UGT74S1* gene amplification

Leaf samples of each 1996 M2 lines and 20 CDC Bethune plants were arranged in individual wells of 21 × 96-well plates containing sterile tungsten beads (Sigma Aldrich, CA, USA), freeze-dried and genomic DNA was extracted using the Qiagen DNeasy Plant Mini Kit (Qiagen, Mississauga, ON) following the manufacturer's recommendations. DNA was quantified using Picogreen (Life technologies, CA, USA) and the quality was verified by agarose gel electrophoresis.

For the amplification and re-sequencing, the genomic DNA region of *UGT74S1* (Supplementary Figures 1A,B) corresponding to locus Lus10017825.g (scaffold35, position 7,827–9,972) of the flax draft genome (Phytozme.jgi.doe.gov) was targeted using universal and standard tagging approaches. The first exon of the gene was amplified and tagged using the universal tagging approach, where the gene specific primers flax1_F1b (**GAAGGTGACCAAGTTCATGC**ACCCCACTTCTCCAATTCTC) and flax1_R1 (**AATGCGTTACAAGCACATCTC**GATTATCATTGCTCGAAACGTG) contained a 5′ tail identical to primers TRS_F (GAAGGTGACCAAGTTCATGC) and TRS_R (AATGCGTTACAAGCACATCTC), respectively. From the TRS_F and TRS_R primers, 96 versions carrying an additional 8-base barcode at the 5′-end were used (Supplementary Table 1). The second exon was amplified and tagged by a more conventional approach using only two primers per PCR reaction. For each gene-specific primer, 96 versions of *UGT74S1* gene-specific primer (flax1_F2b GTCAATCGAGCCAACTCTCG) carrying an additional 8-base barcode at the 5′ end (Supplementary Table 2) were used in combination with gene-specific primer flax1_R2b (ACAAAACCTATCTTCCCAAGTCA). The 20 μl PCR reactions were set up using the 5 × MyTaq Mastermix (Bioline, Berlin, Germany). The standard tagging reactions contained 5 pM of each primer, while the universal tagging reactions contained 2 pM of each gene-specific primer and 20 pM of each TRS primer. Cycling conditions consisted of 2 min at 96°C for pre-denaturation followed by 35 cycles of 15 s at 96°C, 30 s at 55°C, and 2 min at 72°C.

#### Library construction and pyrosequencing

After PCR amplifications, amplicons from each exonic region in each 96-well plate were pooled, purified using MinElute gel purification kit (Qiagen) to remove non-specific amplification products, size-selected and quantified. The two gel-purified amplicon pools derived from each individual exonic DNA plate were mixed in equimolar amounts to create 21 pools, which were further split into a 10 and an 11 pool sets. DNA from each of the 11 or 10 pool sets were ligated to 11 or 10 different 454 multiplex identifier (MID) adaptors and two Roche/454-compatible sequencing libraries were constructed and sequenced by pyrosequencing at LGC Genomics GmbH (Berlin, Germany). All sequencing reactions were based upon FLX-Titanium chemistry (Roche/454 Life Sciences, Branford, CT, USA; www.454.com) and all methods were performed according to manufacturer's protocols. The resulting sequences were processed using the standard Roche software for base calling, adaptor and quality trimming (Genome Sequencer FLX System Software manual version 2.3).

#### Bioinformatics of sequence reads and EMS-induced SNP detection in M2 plants

The sequencing reads were deconvoluted using an in-house-developed tag sorting and clipping program, allowing no more than a single nucleotide mismatch. The barcodes were sorted and trimmed, and individual FASTQ files were generated for each sample.

To confirm the reference identity of the wild type target sequence, reads from the 20 wild type CDC Bethune plants were aligned to the genomic reference sequence of *UGT74S1* (Lus10017825.g) using Bowtie2 v2.1.0 (Langmead and Salzberg, 2012) with default parameters in “local” alignment mode. The alignments were coordinate-sorted and saved in a standard Binary Sequence Alignment/Map (BAM) format. Subsequently, GATK v1.6-11 (http://www.broadinstitute.org/gatk/) was used to call SNPs by running the GATK walkers on the alignment files. Using GATK, IndelRealigner and UnifiedGenotyper were run to realign reads around indels called and to generate raw SNP calls, respectively. To identify and retain high quality SNPs, QualFilter (QUAL ≥ 30.0), QDFilter (QD ≥ 5.0, Quality-by-Depth), DPFilter (DP ≥ 10, sample read depth at locus), StrandFilter (FS ≤ 60.0, Phred-scaled *p*-value using Fisher's Exact Test to detect strand bias), and ABHetFilter (ABHet ≤ 0.9, testing allele balance for heterozygous) were applied using Variant Filtration. The final results were stored in raw and filtered standard variant call format (VCF) files. The VCF files from GATK were further subjected to homopolymer filtering to reduce false-positive calls in homopolymer-dense regions and, this was followed by a final manual inspection of the SNP calls to generate consensus sequences.

To detect EMS-induced SNP in M2 plants, reads from all 1996 M2 EMS lines were aligned using Bowtie2 v2.1.0 as described earlier, and raw and filtered SNPs were called. SNPs causing codon changes were identified using SnpEff v3.2 (http://snpeff.sourceforge.net/). Exon positions described for *UGT74S1* (Ghose et al., 2014) were used to set up a SnpEff database for automatic annotation of VCF files (http://snpeff.sourceforge.net/SnpEff_manual.html#output) and an allele table was constructed for all samples.

A consensus sequence was generated using sequences derived from all samples. Final VCF files from the 1996 M2 lines were used to produce one putative genomic sequence FASTA file per sample. Regions not covered by the PCR amplicons were soft-masked using sequence changed into lowercase, likewise for regions with a read coverage below 10× (for bases with Phred score ≥20). Heterozygous SNP calls were annotated with standard ambiguous nucleotide sequence code (http://www.bioinformatics.org/sms2/iupac.html). The genomic sequences were subsequently used to generate putative coding sequence (CDS) FASTA files and haplotype sequences were generated. Predicted peptide sequences were obtained from the CDS using a standard DNA codon table.

The mutation rate was determined from *UGT74S1* sequence for all the EMS lines sequenced, as performed routinely in EMS TILLING population. The mutation rate in the exonic region was calculated as followed: [(exon 1 + exon 2) × total number of original EMS lines sequenced]/total number of variants observed. Mutational effects for each of the missense and nonsense mutations were predicted using the SuSPect method, a standalone web server (Yates et al., 2014; http://www.sbg.bio.ic.ac.uk/phyre2/html/help.cgi?id=help/investigator). The wild type UGT74S1 protein sequence was uploaded and a heat map was generated for each amino acid position in the protein against a substitution by any of the 20 possible amino acids with color-coded effect level.

### Forward genetics screening for altered SDG lignan phenotypes in M2 families carrying *UGT74S1* EMS-induced mutations

A total of 93 M2 families were identified to carry at least one mutation in *UGT74S1*, of which 18 carried sense mutations and were not considered for further analysis. Among the remaining 75 M2 families carrying EMS-induced missense and nonsense mutations, six had few or no seeds available and could also not be further tested. Because seeds were not collected from the M2 plants grown in the field that were used in reverse genetics, a new set of original M2 seeds were resampled from the remaining 69 M2 families, grown in a greenhouse and subjected to further analyses.

In a first step, forward genetics was performed using a subsample of original M2 seeds from each of the 69 M2 families in which missense or nonsense mutations were detected for assessing the effect of mutations on SDG production. Total lignan was extracted from 50 mg of seeds per family and hydrolyzed by alkaline hydrolysis as described by Ghose et al. (2014). The wild type CDC Bethune was used as control. Non-hydrolyzed lignan polymer and hydrolyzed lignan were purified from families 1230 and 2340 which had nonsense mutations and separated by UPLC to assess the potential effect of mutations on the lignan macromolecule and its constituent phenolic acid glucoside profile. SDG was hydrolyzed from all 69 M2 families, purified, separated by UPLC, confirmed by mass spectrometry and quantitated as previously reported (Ghose et al., 2014). Three independent extractions were performed and analyzed separately. SDG level from each M2 family was compared to that of CDC Bethune.

### Validation of EMS-induced SNPs in M2 plants

After initial SDG lignan determination in the 69 M2 families, we sought to validate the mutations detected by amplicon resequencing in the M2 seeds. Five M2 seeds from each of the 69 M2 families were re-sampled, planted and grown as individual lines in the greenhouse along with CDC Bethune. Each germinated plant was assigned a number and grown to maturity. Hereafter, individual mutant lines are designated by the family number from which they were derived, followed by a number and, if needed, a letter identifier. M3 seeds and leaf tissues were collected separately from each individual M2 plant (or line). At this point, we retained plants (lines) of 28 M2 families with nonsense or missense mutations and that produced enough M3 seeds for further testing (Supplementary Table 3). Genomic DNA was extracted using the Mag-Bind Plant DNA plus Kit (Omega Bio-tek, Norcross, GA, USA) following manufacturer recommendations. SNP re-validation was performed by genotyping 1 or 2 plants (or lines) per family using Kompetitive allele specific PCR (KASP, LGC Genomics, Beverly, MA) assays. A total of 20 KASP assay, were designed to target 16 missense and four nonsense mutations (Supplementary Table 4). Each reaction was carried out using 11–22 technical replicates of each DNA sample and two no template controls to ensure proper clustering as recommended by the supplier. All reactions were performed on a CFX 96 Real Time PCR system (BioRad, Mississauga, ON, Canada) in a total volume of 10 μL containing 5 ng of DNA, 5 μL of 2X KASP master mix, and 0.14 μL of KASP-specific assay mix. The first step of the touchdown PCR included an initial denaturation at 94°C for 15 min, followed by 10 cycles consisting of a denaturation at 94°C for 20 s, a touchdown annealing step starting at 61°C for 60 s and achieving a final annealing at 55°C with 0.6°C decreases per cycle. The second step consisted of 26 cycles, each including a denaturation at 94°C for 20 s and a combined annealing and extension step at 55°C for 60 s. Completed KASP reactions were read at 37°C for 1 min. Two to four additional KASP recycling programs were performed for each assay to improve sample grouping, obtain tighter data point clusters and increase confidence in genotype assignment. A single recycling program consists of three cycles with denaturation at 94°C for 20 s followed by an annealing/extension step at 57°C for 60 s.

### Stability of EMS-induced mutations in M3 plants

To determine the stability and heritability of the EMS-induced mutations confirmed in the M2 flax lines by KASP genotyping, 3–45 viable M3 seeds per M2 plants were planted and grown to maturity in single pots in a greenhouse and from which M4 seeds were harvested. Leaf tissue was collected from each M3 plant, genomic DNA extracted, and KASP genotyping was performed as described above.

### Forward genetics screening for altered SDG lignan phenotypes in M4 plants carrying *UGT74S1* EMS-induced mutations

After re-validation of the EMS-induced mutations in M2 and M3 plants, the stability of changes in the SDG lignan profiles were assessed in the M4 seeds produced by single seed descent of M3 plants derived from eight families (Table 1) as previously described (Ghose et al., 2014).

**Table 1:** M2 families and single seed descent M3 plants with *UGTS74S1* mutations from which M4 seeds were collected and used for phenotypic characterization of SDG lignan content.

**Nos**.	**M2 family ID**	**No. of M3 plants**	**Homozygous**	**Heterozygous**	**Mutation type**
1	1230	3	3	0	Nonsense
2	2004	3	3	0	Missense
3	2092	5	2	3	Missense
4	2340	3	3	0	Nonsense
5	2537	3	1	2	Missense
6	2566	3	0	3	Missense
7	2568	3	3	0	Missense
8	2881	2	2	0	Missense
	Total	25	17	8	

### Confirmation or detection of EMS-induced mutations in *UGT74S1* and other SDG biosynthetic genes using targeted gene panel Ampliseq sequencing

The flax SDG biosynthetic pathway is relatively well-known (Figure 1). To determine whether or not random EMS mutations were also induced in other SDG-related biosynthetic genes of the mutant lines, Ampliseq sequencing of 11 genes, including *UGT74S1*, was performed using the Ion PGM™ system (Life Technologies/Thermo Fischer, CA, USA), as reported by Nishio et al. (2015). The plant material used consisted of a total of 84 M4 plants from 14 lines in six families previously shown to carry homozygous mutations (Supplementary Table 5). Nine CDC Bethune plants were used as positive control. DNA was extracted from each individual plant using the magnetic bead based Mag-Bind Plant DNA Plus 96 extraction kit (Omega Bio-tek) as described earlier and quantified using a Quant-iT PicoGreen dsDNA Assay Kit (Thermo Fisher Scientific, Carlsbad, CA, USA) following manufacturer's instructions.

#### Custom target gene panel design

A custom Ion AmpliSeq™ panel targeting 11 genes, including six dirigent proteins (*DIR 1*–*DIR 6*), two PINO reductases (*PLR* 1 and 2), reported to be involved in SECO biosynthesis (Halls et al., 2005; Nakatsubo et al., 2008; Gasper et al., 2016) and three UGTs (*UGT74S1, UGT74S3*, and *UGT74S4*) recently shown to play different roles in SECO glucosylation (Ghose et al., 2014, Fofana et al., 2017) was designed using Ion AmpliSeq Designer (IAD) software v.2.0.3 (Thermo Fisher Scientific, CA, USA). For each target, the 50 bp upstream and downstream of the genes were added to cover the complete coding sequence. The custom panel was designed in such a way that 11,226 of the 11,248 bp potential targeted regions were covered, accounting for 99.6% coverage (22 bp missing) and included 49 primer pairs divided into two pools of 27 and 22 primer pairs each, respectively (Supplementary Table 6). The targeted 11,248 bp genomic regions were uploaded as a reference BED file sequence. This Ampliseq custom panel was used to screen the 84 EMS mutagenized and nine wild type CDC Bethune plants.

#### Library preparation and sequencing

Genomic DNA and library preparation were performed following Life Technology/Thermo Fisher recommendations. First, pooled barcoded libraries were prepared by pooling equal amount of DNA from three to eight M4 plants from each line per family. Twenty nanograms of genomic DNA was amplified through two multiplex PCR reactions using 27 and 22 sets of Ampliseq primers, respectively (Supplementary Table 6). A total of 12 pooled DNA libraries (11 from mutant lines and one from CDC Bethune) were prepared using the Ion Ampliseq™ Library kit 2.0 (Thermo Fisher Scientific, Carlsbad, CA, USA) according to manufacturer's instructions. Second, individual libraries were prepared from individual M4 plants in each of the lines from which mutations were detected in the pooled libraries. All libraries were quantified using the qPCR based Ion library quantitation kit (Thermo Fisher Scientific), diluted to 30 pM and subjected to sequencing template preparation using the Ion PGM™ Hi-Q™ Chef Kit and the Ion Chef Instrument (Thermo Fisher Scientific) following manufacturer's protocols. Ion 316 and 314 chips were used for loading and sequencing samples of the pooled and individual libraries, respectively. The sequencing was performed using an Ion Torrent Personal Genome Machine (PGM) (Thermo Fisher Scientific). For each run, four libraries were pooled together on each 316-chip, for a minimum of 500× coverage of the 49 amplicons, whereas five individual libraries were pooled together on each 314-chip to achieve a minimum of 100× coverage. All sequencing runs were performed using the Ion PGM HiQ Sequencing kit (Thermo Fisher Scientific) with 850 flows.

#### Mapping sequencing data and variant call

The Ampliseq sequence data was processed using the Ion Suite Software v 5.04 and the Ion Server, and mapped to the flax Ampliseq panel targeted genomic regions uploaded as the reference BED file. Variants were called using Torrent Variant Caller plug-in software set to default parameters under high stringency (minimum allele frequency 0.1, minimum SNP quality 10 and minimum coverage of 5 for SNP identification; minimum allele frequency 0.1, minimum SNP quality 10 and a minimum coverage of 10 for INDEL identification) as performed by Nishio et al. (2015). The selected variant positions were detected, genotyped and reported as wild type, heterozygous or homozygous. For each variant, its mutational effect was predicted using the phyre 2 investigator software as previously reported (Ghose et al., 2015).

## Results

### Targeted amplicons of *UGT74S1*

**Figure 2:**
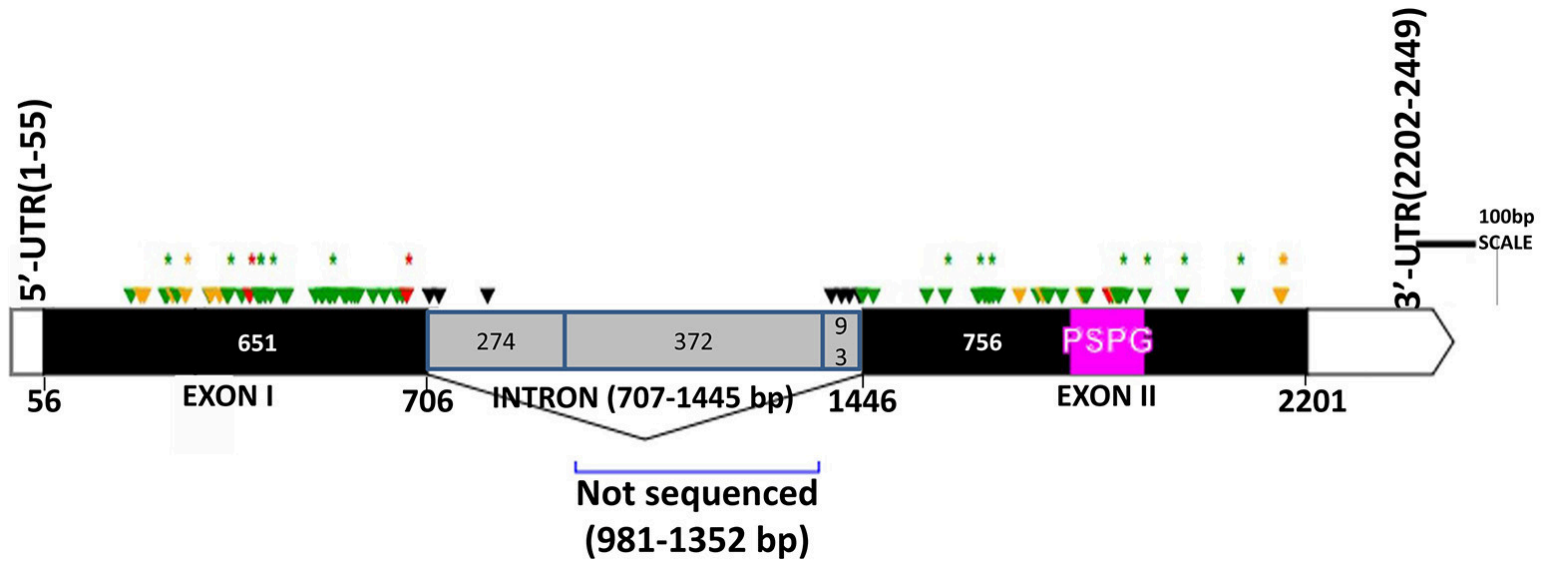
Distribution of EMS-induced mutations within the flax *UGT74S1*. UTRs are indicated by blank boxes, exons by solid black boxes, and the intron by a gray box. The PSPG region within exon 2 is colored in magenta. Green arrows, missense mutations; yellow arrows, sense mutations; red arrows, nonsense mutations; black arrows, mutations in intron; asterisks (^*^) indicate position of homozygous mutations only; Numbers are in bp and the starts and ends of exons are indicated in bp.

The two genomic amplicons containing the *UGT74S1* gene were successfully amplified and sequenced from the 2016 plants. Amplicon 1 included 55 nucleotides of the 5′UTR, the 651 nucleotides of exon 1, and 274 nucleotides of the single intron. Amplicon 2 covered the last 93 nucleotides of the intron, the 756 nucleotides of exon 2, and 248 nucleotides of the 3′UTR. It is worth mentioning that only a portion of the intron, represented by 367 out of the 739 nucleotides of the intronic region was amplified and sequenced (Figure 2).

### EMS-induced mutations detected in *UGT74S1*

Following SNP variants call and filtrations, a total of 80 positions displayed SNP variants, including 40 positions in exon 1, 7 intronic positions, and 33 positions in exon 2 (Table 2). Only 3.12% of the calls were missing for the 80 SNP positions of the 2016 individuals. Since EMS mutagenesis usually causes transition mutations, GC content was determined in the amplicons. Exon 1 had a GC content of 54% and a higher proportion of SNP positions than exon 2 which had a GC content of 46% (Table 2). In the M2 lines sequenced, heterozygosity at SNP positions was observed four times more frequently than homozygosity, showing that most of the EMS-induced mutations were not fixed in the M2 population. Among the exonic mutations, 22.5% were homozygous, thus fixed in these plants. Because not all substitutions have the same mutational effects on gene functionality, the mutational effect for each mutation was determined. Ten mutated amino acids at 12 positions showed high to very high mutational effects, while another 16 amino acid changes were found with low mutational effects at 16 positions (Table 3). Interestingly, two of the mutations with high mutational effects appeared to be nonsense homozygous mutations, thereby expected to have a deleterious effect on SECO glucosylation. Altogether, 73 SNPs were observed in the exonic regions in 93 single plants originating from different M2 families (Figure 2, Table 3). Seven of the 93 M2 plants had two exonic SNPs each (labeled with ^*^ in Table 3). Because six SNP positions had both a homozygous and a heterozygous SNP variants, 79 variants were identified at the 73 exonic SNP positions and, of those, 55 were missense, six were nonsense, and 18 were sense mutations. Only 18 of the 79 SNP variants were homozygous including 13 of the missense, 2 of the nonsense, and 3 sense mutations (Table 3). The mutation rate in the exonic regions was calculated to be 1 per 28 kb [i.e., (651 + 756 bp) × 1996/100 = 28,084 bp/1 or 1 mutation in 28 kb]. Eight of the missense mutations and one nonsense mutation from 12 M2 plants were located in the PSGP region spanning amino acids 334–377 (Figure 2).

**Table 2:** Summary of EMS-induced mutations in *UGT74S1* gene of a mutagenized flax population.

	**Exon 1**	Intron^*^	**Exon 2**
Size	651	367	756
GC (%)	54	37	46
SNP positions	40	7	33
Homozygote SNP count^**^	9	0	9
Heterozygote SNP count^**^	36	7	25

* Only data from the sequenced portion of the intron are reported;

** *Five SNP positions have shown both homozygous and heterozygous SNPs in different lines in Exon 1, for an absolute SNP positions of 40. Similarly, 1 SNP position showed both homozygous and heterozygous SNPs in different lines in Exon 2, for an absolute SNP positions of 33*.

**Table 3:** Details of 79 exonic mutations observed in *UGT74S1* of 93 M2 flax plants.

**SNP Nos**.	**Feature**	**SNP position**	**CDC Beth SNP**	**M2 line SNP**	**Hetero/Homozygote**	**Amino acid position**	**Amino acid change**	**Type of mutation**	**Mutation sensitivity**	**M2 line**
1	exon 1	210	C/C	C/T	Hetero	52	T/I	Missense	Low	2517
2	exon 1	226	C/C	C/T	Hetero	NA	NA	Sense	NA	419
3	exon 1	232	C/C	C/T	Hetero	NA	NA	Sense	NA	1689^*^
4	exon 1	267	G/G	(G/G)/(A/A)	Homo	71	S/N	Missense	Low	2566
	exon 1	267	G/G	G/A	Hetero	71	S/N	Missense	Low	2568
5	exon 1	270	G/G	G/A	Hetero	72	G/E	Missense	Low	686, 692
6	exon 1	280	G/G	G/A	Hetero	NA	NA	Sense	NA	169
7	exon 1	287	C/C	C/T	Hetero	78	P/S	Missense	Medium	1734
8	exon 1	301	C/C	(C/C)/(T/T)	Homo	NA	NA	Sense	NA	730
9	exon 1	341	C/C	C/T	Hetero	96	L/F	Missense	Medium	1864
10	exon 1	343	C/C	C/T	Hetero	96	NA	Sense	NA	2755^*^
11	exon 1	358	C/C	C/T	Hetero	NA	NA	Sense	NA	2436
12	exon 1	370	C/C	C/T	Hetero	NA	NA	Sense	NA	1547
13	exon 1	371	C/C	C/T	Hetero	106	P/S	Missense	Medium	306, 1470
14	exon 1	372	C/C	(C/C)/(T/T)	Homo	106	P/L	Missense	Medium	2092
15	exon 1	395	G/G	G/A	Hetero	114	A/T	Missense	Medium	2729
16	exon 1	408	G/G	(G/G)/(A/A)	Homo	118	W/STOP	Nonsense	Medium	828
	exon 1	408	G/G	G/A	Hetero	118	W/STOP	Nonsense	Medium	879
17	exon 1	422	G/G	(G/G)/(A/A)	Homo	123	A/T	Missense	Medium	27, 31
18	exon 1	423	C/C	(C/C)/(T/T)	Homo	123	A/V	Missense	Medium	217
	exon 1	423	C/C	C/T	Hetero	123	A/V	Missense	Medium	2019^*^
19	exon 1	427	G/G	G/A	Hetero	125	R/R	Sense	NA	2072
20	exon 1	434	G/G	G/A	Hetero	127	G/R	Missense	Low	1523
21	exon 1	443	G/G	G/A	Hetero	130	G/R	Missense	Low	883
	exon 1	443	G/G	(G/G)/(A/A)	Homo	130	G/R	Missense	Low	884
22	exon 1	465	C/C	C/T	Hetero	137	S/L	Missense	Medium	1765
23	exon 1	470	G/G	G/A	Hetero	139	A/T	Missense	Medium	1312
24	exon 1	518	C/C	C/T	Hetero	155	P/S	Missense	Low	2410
25	exon 1	528	C/C	C/T	Hetero	158	S/L	Missense	Low	936
26	exon 1	530	G/G	G/A	Hetero	159	D/N	Missense	Medium	2200
27	exon 1	540	C/C	C/T	Hetero	162	A/V	Missense	Low	184
28	exon 1	542	G/G	G/A	Hetero	163	G/R	Missense	Low	756
29	exon 1	543	G/G	(G/G)/(A/A)	Homo	163	G/E	Missense	Low	1455
30	exon 1	550	C/C	C/T	Hetero	NA	NA	Sense	NA	685, 1646
31	exon 1	566	C/C	C/T	Hetero	171	P/S	Missense	Medium	575
32	exon 1	570	C/C	C/T	Hetero	172	P/L	Missense	Medium	2019^*^
33	exon 1	578	G/G	G/A	Hetero	199	S/F	Missense	Low	2551
34	exon 1	584	G/G	G/A	Hetero	177	D/N	Missense	Medium	937
35	exon 1	589	G/G	G/A	Hetero	NA	NA	Sense	NA	65, 66
36	exon 1	614	C/C	C/T	Hetero	187	H/Y	Missense	Medium	878
37	exon 1	633	C/C	C/T	Hetero	193	A/V	Missense	Low	758
38	exon 1	654	A/A	A/C	Hetero	200	N/T	Missense	Low	559
39	exon 1	663	C/C	C/T	Hetero	203	A/V	Missense	Medium	731
40	exon 1	670	G/G	G/A	Hetero	205	W/STOP	Nonsense	Medium	919, 1767^*^, 1885
	exon 1	670	G/G	(G/G)/(A/A)	Homo	205	W/STOP	Nonsense	Medium	1230
41	exon 2	1447	C/C	C/T	Hetero	218	A/V	Missense	Medium	1592
42	exon 2	1465	A/A	A/G	Hetero	224	K/R	Missense	Low	132
43	exon 2	1554	A/A	A/G	Hetero	254	I/V	Missense	Medium	606
44	exon 2	1585	G/G	(G/G)/(A/A)	Homo	264	C/Y	Missense	High	2881
45	exon 2	1639	G/G	(G/G)/(A/A)	Homo	282	G/E	Missense	High	2004, 2010
46	exon 2	1642	G/G	G/A	Hetero	283	S/N	Missense	Very High	1616
47	exon 2	1651	G/G	G/A	Hetero	286	R/K	Missense	Medium	2501
48	exon 2	1657	C/C	C/T	Hetero	288	S/L	Missense	Medium	698
49	exon 2	1659	C/C	(C/C)/(T/T)	Homo	289	P/S	Missense	Medium	2229
50	exon 2	1665	C/C	C/T	Hetero	291	Q/STOP	Nonsense	High	1777
51	exon 2	1674	G/G	G/A	Hetero	294	E/K	Missense	High	12^*^, 2800, 2801^*^
52	exon 2	1709	C/C	C/T	Hetero	NA	NA	Sense	NA	1645
53	exon 2	1740	G/G	G/A	Hetero	316	A/T	Missense	Low	2067
54	exon 2	1748	C/C	C/T	Hetero	NA	NA	Sense	NA	604
55	exon 2	1755	G/G	G/A	Hetero	321	E/K	Missense	Medium	12^*^, 2801^*^
56	exon 2	1758	G/G	G/A	Hetero	322	E/K	Missense	Medium	2741, 2755^*^
57	exon 2	1780	G/G	G/A	Hetero	329	G/E	Missense	High	1291, 1292, 1309, 1453^*^, 1555
58	exon 2	1814	C/C	C/T	Hetero	NA	NA	Sense	NA	2413
59	exon 2	1815	C/C	C/T	Hetero	NA	NA	Sense	NA	366
60	exon 2	1818	G/G	G/A	Hetero	**342**	A/T	Missense	NA	1689^*^
61	exon 2	1822	G/G	G/A	Hetero	**343**	S/N	Missense	Medium	1427
62	exon 2	1859	G/G	G/A	Hetero	**355**	W/STOP	Nonsense	Very high	2340
63	exon 2	1864	C/C	C/T	Hetero	**357**	S/L	Missense	Very high	614
64	exon 2	1869	C/C	C/T	Hetero	**359**	L/R	Sense	Medium	1453^*^, 1635
65	exon 2	1872	G/G	G/A	Hetero	**360**	E/K	Missense	Very high	2525, 2537
66	exon 2	1875	G/G	G/A	Hetero	**361**	A/T	Missense	Medium	1767^*^
67	exon 2	1878	C/C	(C/C)/(T/T)	Homo	**362**	L/F	Missense	High	1571, 1572
68	exon 2	1887	G/G	G/A	Hetero	**365**	G/R	Missense	High	463, 2526
69	exon 2	1918	G/G	(G/G)/(A/A)	Homo	**375**	G/D	Missense	High	1403
70	exon 2	1980	G/G	(G/G)/(A/A)	Homo	396	A/T	Missense	Medium	882
71	exon 2	2074	G/G	(G/G)/(A/A)	Homo	427	R/K	Missense	Medium	250
72	exon 2	2144	C/C	C/T	Hetero	NA	NA	Sense	NA	557, 567
	exon 2	2144	C/C	(C/C)/(T/T)	Homo	NA	NA	Sense	NA	573
73	exon 2	2147	G/G	(G/G)/(A/A)	Homo	NA	NA	Sense	NA	2398

NA, Not applicable for no amino acid change;

* *Lines with mutations at two loci. Heterozygous SNP (single strand nucleotide change) and homozygous SNP (change of double strand nucleotide as marked in bracket) at each position are indicated and separated by a “/”. Positions of amino acids changed by EMS mutagenesis within the PSPG motif are indicated in bold*.

### Effect of *UGT74S1* mutations on SDG content in bulked M2 seeds

**Figure 3:**
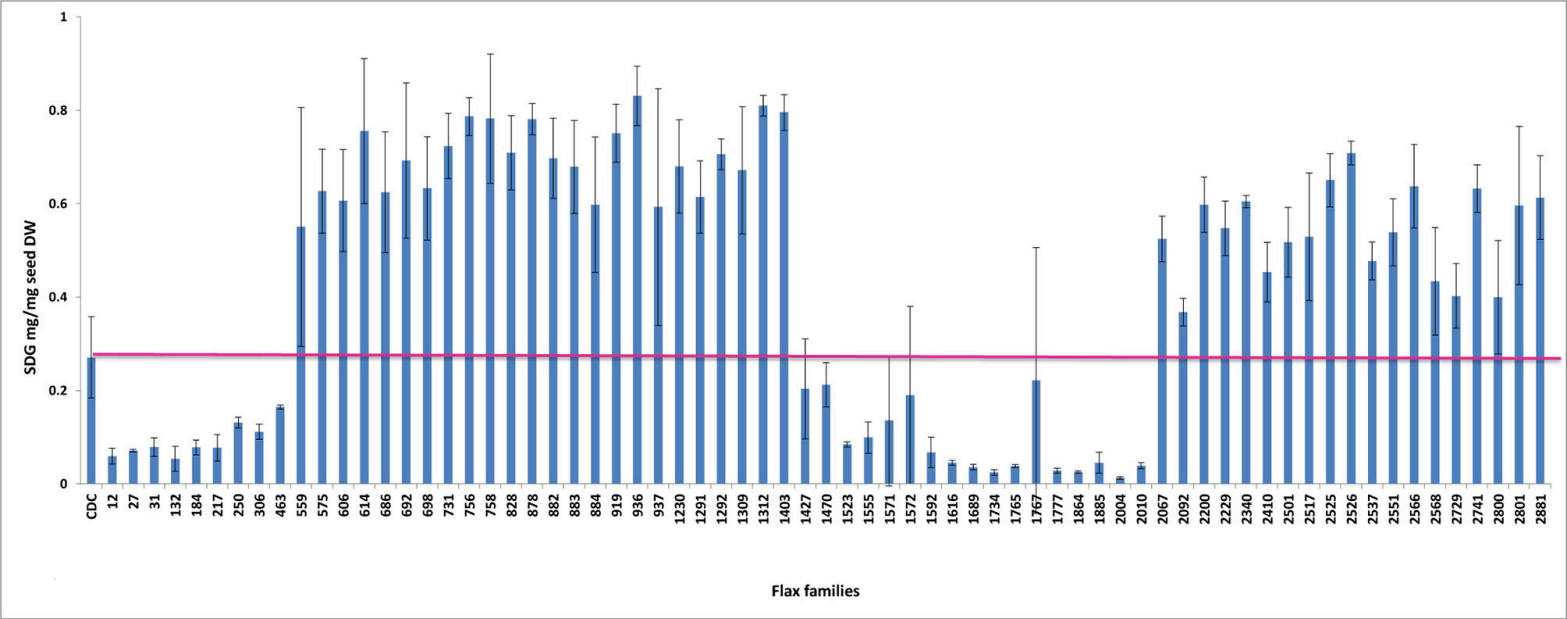
Diversity in the spectrum of SDG production in wild type Bethune (CDC) and 69 EMS mutant lines. The pink line indicates the CDC Bethune threshold line for comparison.

To establish a genotype to phenotype relationship, the effect of each mutation on SDG lignan production was assessed *in planta* by determining the total SDG lignan content in the original mature bulked M2 seeds in comparison with that of the wild type CDC Bethune. UPLC chromatograms of non-hydrolyzed oligomeric lignan polymers from the M2 family 1230 carrying a homozygous nonsense mutation in *UGT74S1* displayed a narrower peak whereas that of the M2 family 2340 that carried a heterozygous nonsense mutation showed additional peaks overlapping the main peak (Supplementary Figure 2A). Variations were also observed in the profiles of SDG and other phenolic acid glycosides present in the lignan macromolecule (Supplementary Figure 2B). By determining the total hydrolyzed SDG lignan content in the original M2 bulked mature seeds from each of the 69 M2 families, we observed 21 with reduced SDG content, eight of which displayed almost no SDG. In contrast, 43 families had a higher SDG content compared to CDC Bethune (Figure 3). In many cases, no direct correlation could be established between the mutation and alteration of the lignan profile. In fact, some M2 families such as 828, 919, 1230, and 2340, carrying homozygous nonsense mutations showed increased SDG lignan content compared to the wild type. Similarly, eight M2 families (882, 1403, 1571, 1572, 2092, 2229, 2566, and 2881) carrying homozygote missense mutations showed higher or inconsistent lignan content (Figure 3). These unexpected observations prompted us to re-assess and re-validate the genotype of single M2 plants derived from each of the 69 M2 families after re-sampling within the original seed lots.

### Validation of EMS-induced mutations in M2 plants

To validate some of the EMS-induced mutations identified in *UGT74S1* by amplicon resequencing, plants from 28 M2 families (Supplementary Table 3) were genotyped by KASP assays (LGC Genomics, UK) using markers targeting 20 EMS-induced SNP mutations. Ten of the 20 targeted mutations were thus validated in 21 plants from 15 M2 families, but the other 10 from 13 M2 families were not (Table 4). Among the validated plants, 17 were heterozygous in 13 families and four plants from four different families were homozygous. After validation of EMS-induced mutations in the M2 plants, the stability of the mutations was determined in single seed descent M3 plants derived from KASP-validated M2 plants. KASP assays showed that all M3 plants derived from homozygous M2 plants remained homozygous whereas M3 plants derived from a heterozygous M2 plants were either heterozygous or segregated (Table 4).

**Table 4:** Mutation stability and segregation among seed from single flax bolls.

**M2 family ID**	**Target SNP**	**CDC Beth**	Original M2^*^ plant genotype	Number of M2 plants tested by KASP^**^	**M2 KASP genotype**	**M2 KASP validation**	**Number of M3 plants**	**M3 KASP genotype**	**M3 KASP validation**	**Genotype status**
12	1674	G/G	G/A	2	G/G	No	3	3 G/G	No	Homo wild
	1755	G/G	G/A	2	G/G	No	NA	NA	NA	NA
306	371	C/C	C/T	2	C/C	No	NA	NA	NA	NA
463	1887	G/G	G/A	2	G/G	No	NA	NA	NA	NA
756	542	G/G	G/A	1	G/G	No	6	6 G/G	No	Homo wild
828	408	G/G	A/A	2	G/G	No	27	27 G/G	No	Homo wild
919	670	G/G	G/A	1	G/A	Yes	25	25 G/A	Yes	Heterozygous
936	528	C/C	C/T	1	C/C	No	8	8 C/C	No	Homo wild
1230	670	G/G	A/A	2	G/A	Yes	21	3 G/G; 13 G/A; 5 A/A	Yes	Segregate
1427	1822	G/G	G/A	2	G/G	No	10	10 G/G	No	
1470	371	C/C	C/T	2	C/C; C/T	Yes	34	2 C/T; 32 C/C	Yes	Segregate
1689	1818	G/G	G/A	2	G/G	No	4	4 G/G	No	Homo wild
1767	670	G/G	G/A	1	G/A	Yes	19	19 G/A	Yes	Heterozygous
	1875	G/G	G/A	NA	NA	NA	NA	NA	NA	NA
1777	1665	C/C	C/T	1	C/C	No	28	28 C/C	No	Homo wild
1885	670	G/G	G/A	2	G/A	Yes	27	26 G/A; 1 G/G	Yes	Segregate
2004	1639	G/G	A/A	1	A/A	Yes	27	27 A/A	Yes	Homo stable mutant
2010	1639	G/G	A/A	2	G/G	No	17	17 G/G	No	Homo wild
2092	372	C/C	T/T	2	T/T; C/T	Yes	20	2 T/T; 5 C/T; 13 C/C	Yes	Segregate
2340	1859	G/G	G/A	2	A/A; G/A	Yes	29	16 A/A; 12 G/A; 1 G/G	Yes	Segregate
2517	210	C/C	C/T	2	C/C	No	NA	NA	NA	NA
2525	1872	G/G	G/A	2	G/A	Yes	24	7 A/A; 7 G/G; 10 G/A	Yes	Segregate
2526	1887	G/G	G/A	1	G/A	Yes	18	9 G/A; 8 G/G; 1 A/A	Yes	Segregate
2537	1872	G/G	G/A	2	G/A	Yes	40	35 G/A; 1 G/G; 4 A/A	Yes	Segregate
2566	267	G/G	A/A	1	G/A	Yes	45	20 G/G; 7 G/A; 18 A/A	Yes	Segregate
2568	267	G/G	G/A	2	G/G; G/A	Yes	23	13 G/G; 3 G/A; 7 A/A	Yes	Segregate
2741	1758	G/G	G/A	2	G/G	No	7	7 G/G	No	Homo wild
2800	1674	G/G	G/A	2	G/G; G/A	Yes	NA	NA	NA	NA
2801	1674	G/G	G/A	2	G/G	No	12	12 G/G	No	Homo wild
2881	1585	G/G	A/A	1	A/A	Yes	23	23 A/A	Yes	Homo stable mutant

Genotypes showing identical alleles to the wild type CDC Bethune are wild type homozygous; genotypes with an alternate allele are heterozygous mutants, genotypes with two identical alleles but different from wild type alleles are homozygous mutants.

* Genotype of original M2 plants used in amplicon sequencing. These plants were different from those used in KASP genotyping although they all came from the same M2 seed lot;

** *M3 plants and M4 plants were subsequently derived from these M2 plants; NA, not applicable meaning no further testing*.

### Stability of EMS-induced mutation effect on SDG content in M4 seeds derived from single seed descent of M3 plants

**Figure 4:**
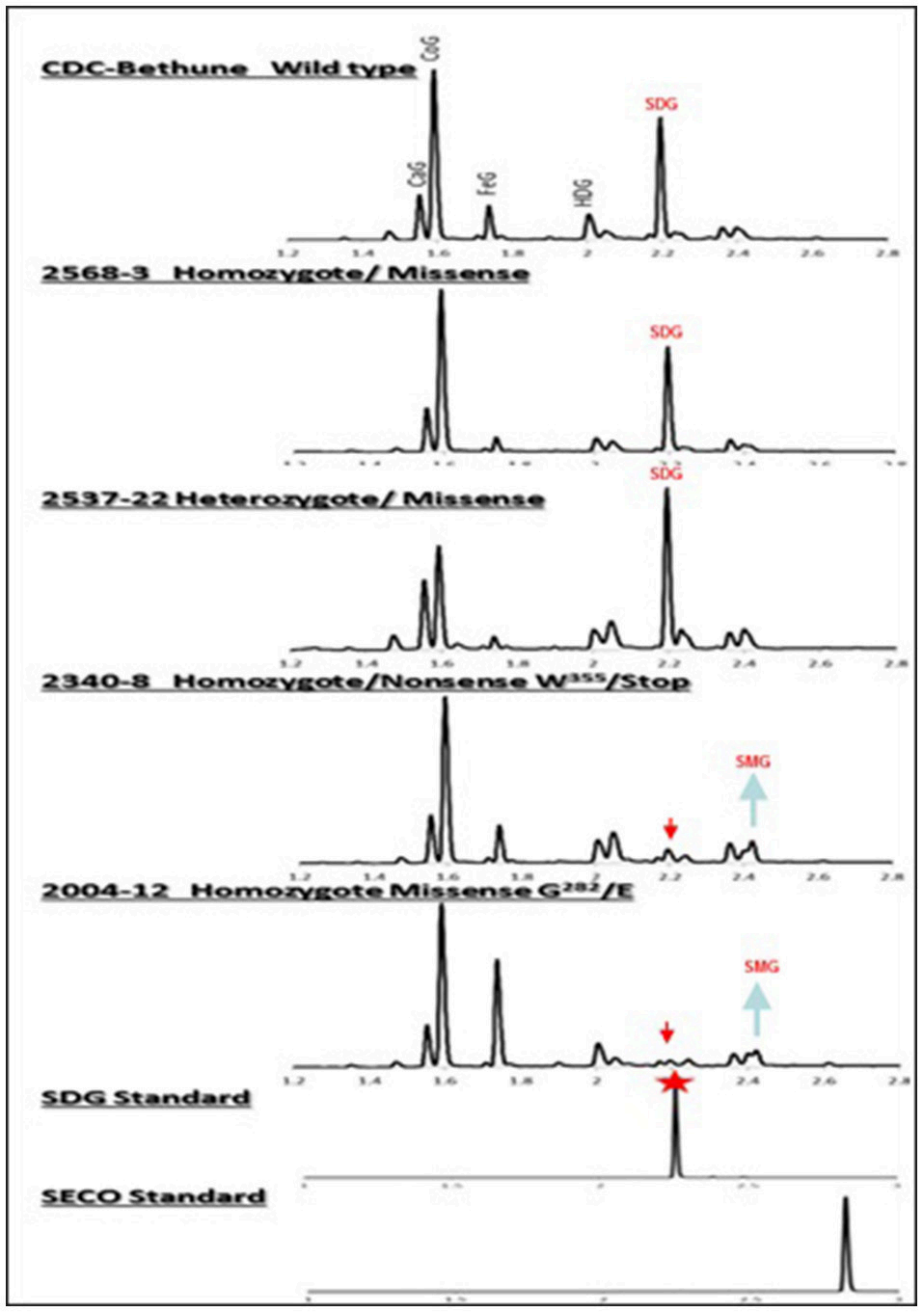
UPLC chromatograms of hydrolyzed lignan complex from M4 seed derived from M3 single seed descent lines and seed from CDC Bethune showing the diversity in SDG profile. The peaks of phenolic acid glucosides and SDG are shown on the top. CaG, caffeic glucoside; CoG, coumaric glucoside; FeG, ferulic glucoside; HDG, herbacetin diglucoside. The red arrows indicate the disappearance of SDG peaks in homozygote nonsense (#2340) and missense (#2004) M4 lines compared to CDC Bethune. Note the equal and higher SDG peaks in the seed from homozygous (#2568) and heterozygous (#2537) lines carrying missense mutations. SDG peak from the SDG standard is indicated by the red star.

**Figure 5:**
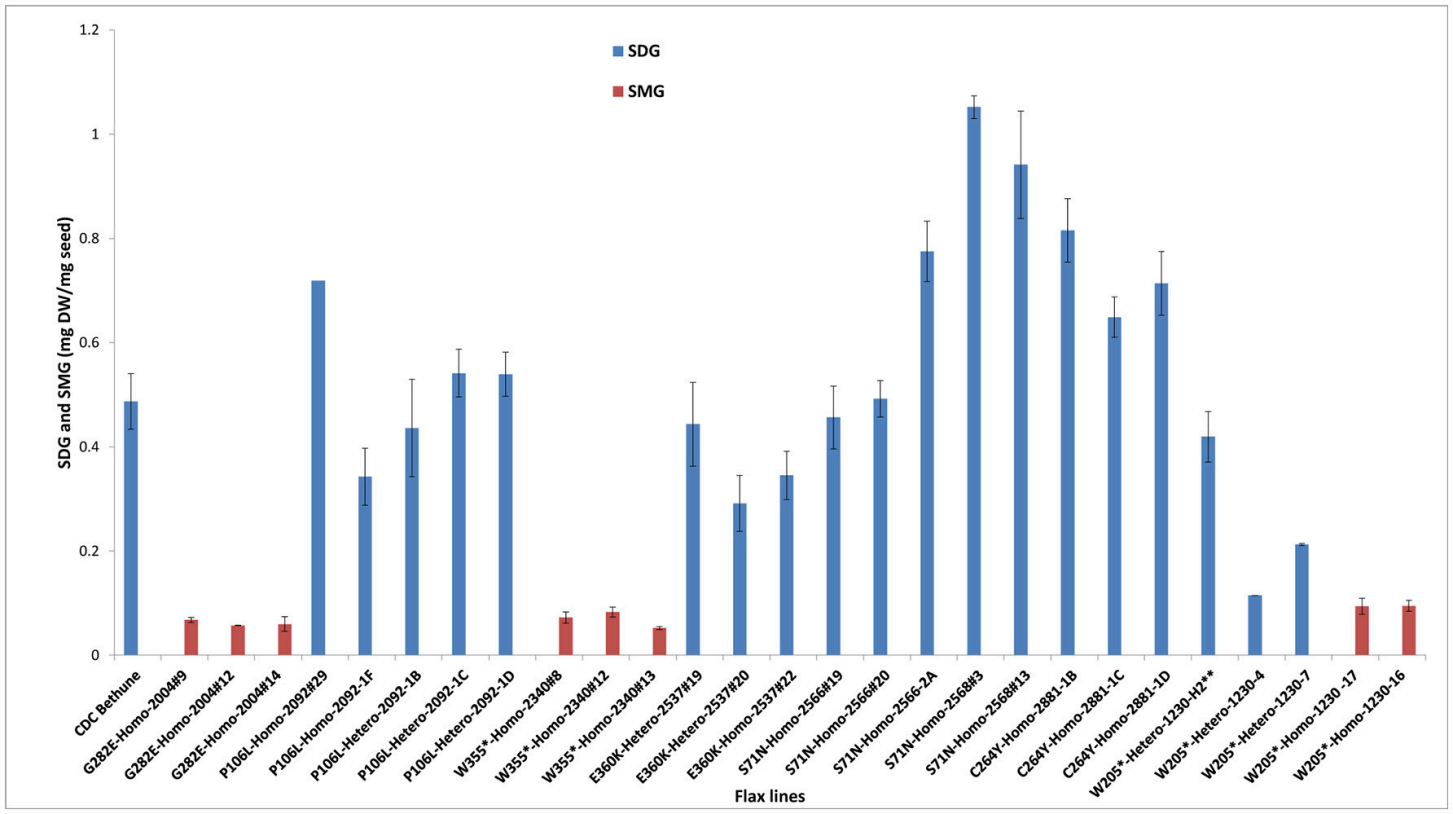
Variations in SDG and SMG lignan contents in M3 and M4 seeds from EMS mutagenized flax lines following lignan complex hydrolysis. SDG and SMG UPLC peaks were monitored using mass spectra and quantified. EMS lines are named as EMS-induced amino acid changed followed by the homo or heterozygous status and the line number. Single asterisk (^*^) indicates premature stop codon. The profile of M3 seeds derived from M2 line 1230-H2 is indicated by a double asterisk (^**^). Lignan was extracted from CDC Bethune and from M4 seeds derived from their respective M3 plants.

By phenotyping the M4 seeds derived from individual M3 plants for SDG, we identified knock-out mutants devoid of SDG (Figure 4). However, most of the heterozygous and homozygous missense mutations showed a wide range of SDG content, some having SDG content greater than CDC Bethune and others, such as M4 plants from family 2004 (homozygous missense mutation) were complete knock-outs (Figure 5). Surprisingly, disappearance of the SDG peak did not result in the appearance of SECO peaks in any of the knock-out lines. Instead, the mass spectra analysis of peak #10 revealed the presence of SMG in these knock-out lines but not in the others (Figure 5). Interestingly, EMS mutations led to variations in the levels of the phenol acid glucosides encountered in the lignan macromolecule (Supplementary Figure 3).

### *UGT74S1* is unique in controlling SECO glucosylation into SDG

**Figure 6:**
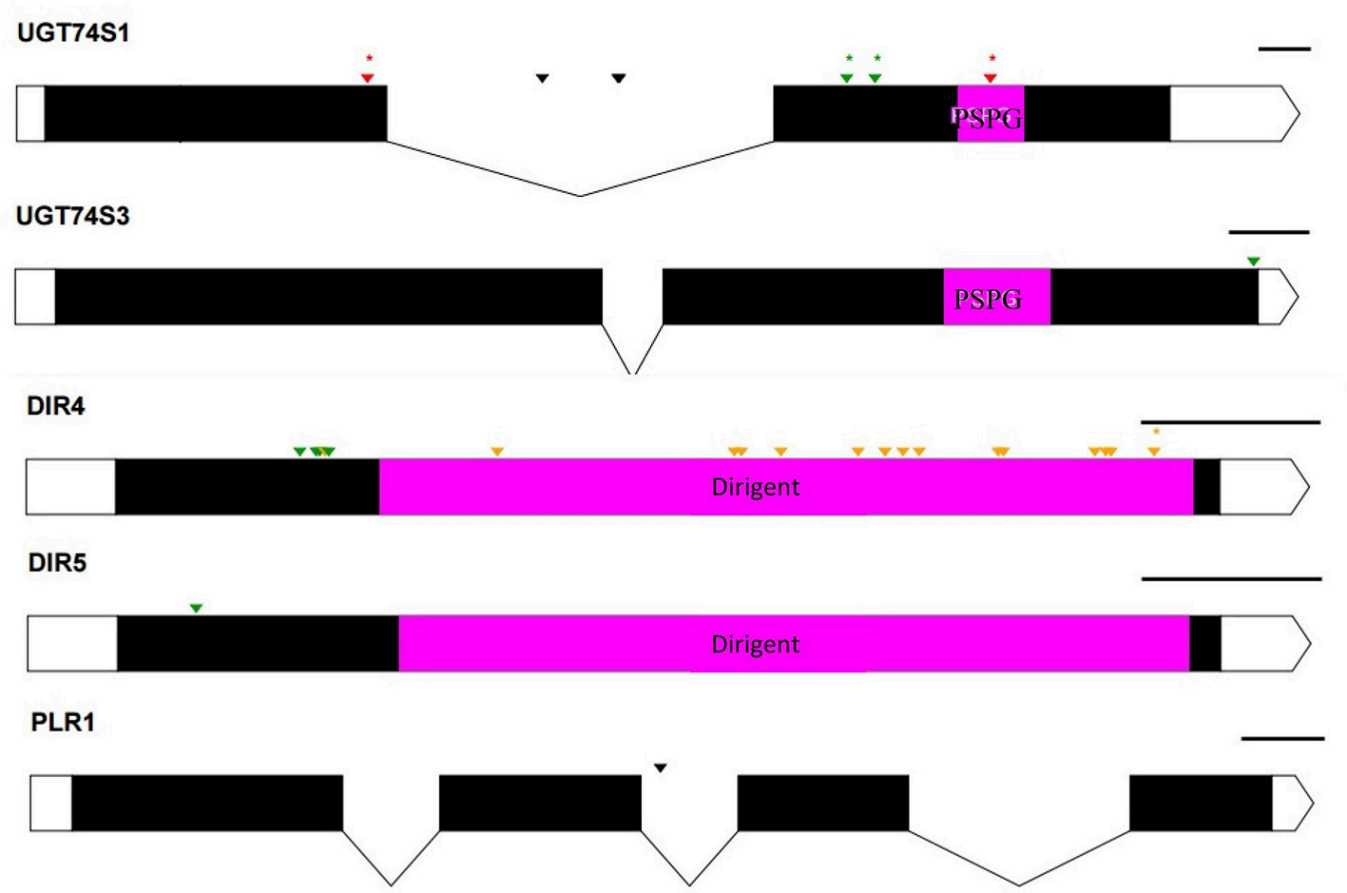
Distribution of EMS-induced mutations on five genes of the 11 custom gene panel submitted to Ampliseq sequencing. UTRs are indicated by white boxes, exons by solid black boxes, and introns by gaps with downward arrows. The PSPG region within exon 2 of UGT74S1 and UGT74S3 is colored in magenta. The magenta box in DIR genes indicates DIR protein domains. The PSPG and dirigent regions are conserved domains for UGT and dirigent proteins, respectively. Green arrows, missense mutations; orange arrows, sense mutations; red arrows, nonsense mutation; black arrows, mutations in introns; asterisks (^*^) indicate position of homozygous mutations only; The black lines on the right top of each gene indicate the gene drawing scale.

To ascertain whether mutations occurred in other lignan-related biosynthetic genes that may have contributed to the observed altered lignan phenotypes, plants with altered lignan profiles were subjected to targeted Ampliseq gene sequencing of 11 genes known to play diverse roles in SDG lignan biosynthesis (Figure 1). For each gene sequenced, a high percentage of aligned bases (99.2%) was achieved, with an average coverage depth of 5,340×. Of the six M4 flax families used in the Ampliseq sequencing experiment, all plants from lines in four of the families were re-confirmed with the mutations previously detected in *UGT74S1* by amplicon sequencing and confirmed by KASP genotyping. No loss-of-function mutation was found in any plants of the lines derived from families 2566 and 2568. A mutation with low sensitivity was detected only in plants from lines #2004-9 and #2004-14 at SNP position 1,537 of *UGT74S3* that caused the *P471S* missense mutation with potential neutral effect. No mutation was detected in *UGT74S4* in any of the plants. Similarly, no mutation was found in any of the three *UGTs* (*UGT74S1, UGT74S3*, and *UGT74S4*) of plants derived from CDC Bethune. In contrast, three mutations (*Y35H, K38H, R40Q*) and one (*L15S*) with low mutational sensitivity were detected in *DIR 4* and *DIR 5*, respectively, in M4 plants of lines# 2004-9 and 2004-14 (Figure 6, Table 5, Supplementary Table 7).

**Table 5:** Mutations confirmed in *UGT74S1* or identified in other genes by Ampliseq sequencing.

** 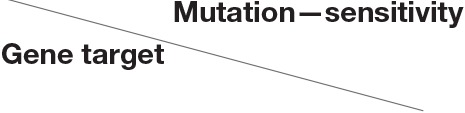 **	***W205/Stop* medium**	***W355/Stop* very high**	***C264Y* High**	***G282E* High**	***P471S* Low**	***Y35H*—*K38H*—*R40Q* Low**	***L15S* Low**
*UGT74S1*	1230-16	2340-8	2881-1B	2004-9	NA	NA	NA
	1230-17	2340-12	2881-1C	2004-12			
		2340-13	2881-1D	2004-14			
*UGT74S3*	NA	NA	NA	NA	2004-9	NA	NA
					2004-14		
*DIR4*	NA	NA	NA	NA	NA	2004-9	
						2004-14	
*DIR5*	NA	NA	NA	NA	NA	NA	2004-9
							2004-14

*Lines in which mutations were found are shown and indicated by EMS family number (four Arabic numbers) followed by a dash and line numbers (1 or 2 digits arabic or alpha numeric numbers). NA, not applicable: indicates that no such mutation was found in that gene. The two lines 2004-9 and 2004-14 from family 2004 and carrying mutations in more than one genes are highlighted in red*.

## Discussion

Establishing phenotype to genotype relationship and assigning functions to variant alleles are of interest to plant breeders and biologists. Using chemically induced mutations and TILLING as a reverse genetics tool, gene and allele functions have been elucidated in several biosynthetic pathways in plants (Slade and Knauf, 2005; Till et al., 2007; Xin et al., 2008; Dahmani-Mardas et al., 2010; Uchida et al., 2011; Rawat et al., 2012; Chantreau et al., 2013; Dhaliwal et al., 2015; Galindo-González et al., 2015). In the current study, we mined an EMS mutagenized flaxseed population and used an NGS-based reverse genetics platform to identify flax lines carrying mutations in the lignan glucosylation gene *UGT74S1* and to determine allele-specific mutational effects on SDG lignan production *in planta*. The study demonstrated that EMS-induced mutations in *UGT74S1* resulted in alteration of SDG accumulation in seeds and, as such, it constitutes the first report of non-transgenic flax germplasm with altered lignan content.

Whereas, TILLING terminology is relatively new, generating mutant alleles in flax using EMS is not (Green and Marshall, 1984; Rowland et al., 1995). The novelty of the chemical mutagenesis in the plant reverse genetics field resides in the much larger screening throughput capacity that we can now achieve (Sikora et al., 2011; Vicente-Dólera et al., 2014). In the present report, 1996 M2 families were screened by reverse genetics and, as expected, only G to A or C to T transition mutations were observed (Sikora et al., 2011). The calculated mutation frequency of one mutation per 28 kb is close to the range recently reported rate of one mutation per 30–49 kb in flax treated with different doses of EMS (Chantreau et al., 2013). A high proportion of 91% of the SNP variation was located in exons, in agreement with their higher G/C content which are more prone to EMS alkylation causing mainly transition mutations (Sikora et al., 2011). In this study, 22.5% of exonic mutations were homozygous, with potential loss (Uchida et al., 2011) or gain-of-function effects (Bailly et al., 2014). Indeed, whereas DNA mutations can affect any of the amino acids, not all substitutions have the same mutational effects on gene functionality (Rogers et al., 2000). Moreover, depending on the amino acids, homozygous missense mutations showing low to medium mutational effects can be expected to be neutral or hypermorphic. In the latter, the mutation may cause an increased contribution of the mutated gene to the original function (Lu et al., 2012).

By genotyping three generations of plants, the identified EMS-induced mutations were heritable, stable, or segregating, as reported in other plants (Uauy et al., 2009). Through phenotyping we showed that nonsense and missense mutations in *UGT74S1* coding regions altered SDG lignan and phenolic acid glucoside profiles in flax seed. In a previous study, we showed that site-directed mutagenesis of targeted amino acids within the PSPG (W^334^–Q^374^) motif of UGT74S1 abolished completely or altered the SECO glucosylation *in vitro* (Ghose et al., 2015). In agreement with these findings, the EMS treatment randomly mutagenized nine amino acids (*A342F, S343N, W355Stop, S357L, E360K, A361T, L362F, G365R*, and *G375D*) within the PSPG *in planta* and, as previously observed *in vitro*, homozygous nonsense EMS-induced mutation of W^355^ to stop codon completely abolished SDG formation *in planta*, but retained trace amounts of SMG. Homozygous nonsense mutation of W^205^ to a stop codon outside the PSGP as well as the homozygous missense *G282E* mutation also led to loss-of-function of UGT74S1 for SECO glucosylation into SDG, but again a small amount of SMG remained. In a recent study, we reported that *UGT74S1* is the key player in controlling SECO glucosylation into SDG, while also showing that *UGT74S3* and *UGT74S4*, a pair of duplicated genes most closely related to *UGT74S1*, were able to glucosylate SECO into SMG at low efficiency but unable to synthesize SDG from SMG (Fofana et al., 2017). These findings suggested that these two paralogs may play roles in other biological processes other than SDG formation from SECO (Fofana et al., 2017). This was corroborated herein where we showed that plants from four *UGT74S1* EMS lines lacking mutations in *UGT74S3* and *UGT74S4* failed to produce SDG but could still produce trace amounts of SMG. This clearly demonstrates occurrence of low background activity of UGT74S3 and UGT74S4 catalyzing trace SMG formation despite UGT74S1 being knocked out, in agreement with our previous report (Fofana et al., 2017). Moreover, some residual glucosylation activity may also be associated with each amino acid substitution effect within the mutated *UGT74S1*, as reported by *in vitro* site-directed mutagenesis data (Ghose et al., 2015). Additionally, the pool of phenolic acid glucosides was found to be increased in all mutant lines, as compared to the lignan component of the polymer. Additional biochemical studies using the phenolic acids encountered in the lignan macromolecule as substrates are required to characterize the exact role(s) of these two UGT paralogs *in planta*.

Targeted gene panel sequencing by Ampliseq methodology confirmed the mutations detected in *UGT74S1* by amplicon re-sequencing and KASP assays. It also identified the *P471S* mutation in *UGT74S3* from *UGT74S1* mutant lines G282E-Homo-2004-9 and G282E-Homo-2004-14, each showing small amounts of SMG, thereby demonstrating the residual activity from UGT74S4 and/or the residual glucosylation activity associated with the G282E substitution in the mutated UGT74S1. The M4 plants from lines 2004-9 and 2004-14 also carried mutations, albeit with low effects, in both *DIR4* and *DIR5* genes at positions *Y35H, K38H, R40Q*, and *L15S*, but still produced trace amounts of SMG. This observation demonstrates that SECO was produced in these lines and that DIR4 and DIR5 are not rate limiting in the pinoresinol formation. Considering that not all the flax genome was scanned in the current study, one may wonder whether some transcription factors controlling lignan biosynthetic genes may have also been affected by EMS. Whereas, this assumption cannot be totally ruled, it seems to be highly unlikely or these mutations, if any, may be of very low or neutral effect because the lignan phenotypes observed in the lines herein described are in agreement with that from the *in vitro* data (Ghose et al., 2015), in the absence of implication of any transcription factors.

In conclusion, this study found EMS mutagenesis to be successful in altering SECO glucosylation toward SDG formation *in planta*. The wide range of lignan profiles observed from the current investigation constitutes a useful non-GMO genetic resource for flax breeders in developing new cultivars with high SDG content or new cultivars with SMG as a new trait in flax. It also offers plant biologists and natural health product scientists an opportunity to better understand the behavior of plants carrying the new trait in field environments. Using Ampliseq sequencing of *UGT74S1* along with 10 other genes potentially playing a role in the SDG production, we validated our SNP detection approach and provided further evidence that UGT74S3 and UGT74S4 are not critical for SDG production *in planta* and that *UGT74S1* is unique in controlling SDG formation from SECO.

## Author contributions

BF: Conception, coordination, design, experiments, data analysis, interpretation, and writing of the manuscript; KG: Performed experiments, data analysis, interpretation, drafting and revision of the manuscript; JM: UPLC and mass spectrometry data acquisition and analysis of lignan extracts, revision of manuscript; DM and AS: EMS population nursery; SC: coordination, administration, and revision of the manuscript; MD and GR: EMS mutagenesis and M2 population generation, revision and proof reading of the manuscript. All authors read, commented and approved the manuscript.

### Conflict of interest statement

The authors declare that the research was conducted in the absence of any commercial or financial relationships that could be construed as a potential conflict of interest.

## Supplementary material

Supplementary Figure 1 Reverse genetics of 1996 EMS-mutagenized M2 flax lines targeting *UGT74S1* gene. **(A)** genomic region of *UGT74S1* used for targeted amplicon resequensing; **(B)** Schematic representation of the workflow adopted for SNP variants detection in the EMS flax population.

Supplementary Figure 2 UPLC chromatograms of non-hydrolyzed **(A)** and hydrolyzed **(B)** lignan extracted from bulked seeds of wild type CDC Bethune and M2 families 1230 and 2340, identified to carry nonsense mutations in *UGT74S1*. **(A)** lignan macromolecule profiles for the wild type CDC Bethune, M2 family 1230 predicted to have a premature stop codon at amino acid 205, and M2 family 2340, heterozygous for SNP-1859 and predicted to encode a wild type allele of *UGT74S1* gene and a mutant one with a premature stop codon at amino acid 355. Note the narrower width of the lead peak in family 1230 compared to CDC Bethune as indicated by the two arrows and additional peaks on the lead peak in family 2340 as indicated by stars compared to CDC Bethune and M2 family 1230. The patterns of both M2 families also displayed additional and higher peaks (indicated by stars) in other parts of the chromatograms compared to CDC Bethune. **(B)** UPLC chromatograms of the hydrolyzed lignan complex from CDC Bethune, M2 families 1230 and 2340 showing peak height variations for SDG and phenolic acid glucosides encountered in the complex lignan polymers.

Supplementary Figure 3 Summary of mutational effects on lignan glucoside species production in *UGT74S1* flax mutant lines. Number of stars represents the relative amount of lignan species; HCA, hydroxycinnamic acid.

## Abbreviations

SECO: Secoisolariciresinol
SDG: Secoisolariciresinol diglucoside
SMG: Secoisolariciresinol monoglucoside
PINO: Pinolariciresinol
ENL: Enterolactone
END: Enterodiol
UGT: Uridine glucosyl transferases
PSPG: Plant secondary product glucosyl transferase
EMS: Ethyl methane sulphonate
KASP: Kompetitive allele specific PCR
SNP: Single nucleotide polymorphism
UTR: Untranslated region
CDS: Coding DNA sequence
ER: Estrogen receptor
VCF: Variant call format.
