# Supplementary figures and images for: Induced Mutagenesis in UGT74S1 Gene Leads to Stable New Flax Lines with Altered Secoisolariciresinol Diglucoside (SDG) Profiles
Source: Front Plant Sci. 2017 Sep 21;8:1638. doi: 10.3389/fpls.2017.01638 (PMC5613138; doi:10.3389/fpls.2017.01638)

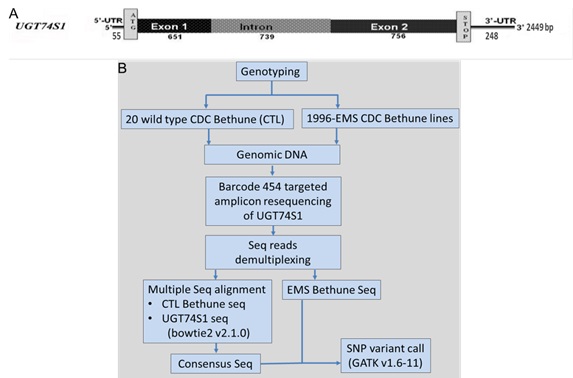

Supplement: Supplementary Figure 1 — Reverse genetics of 1996 EMS-mutagenized M2 flax lines targeting UGT74S1 gene. (A) genomic region of UGT74S1 used for targeted amplicon resequensing; (B) Schematic representation of the workflow adopted for SNP variants detection in the EMS flax population. [file Image1.JPEG]

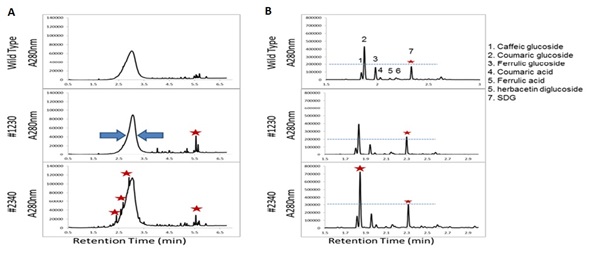

Supplement: Supplementary Figure 2 — UPLC chromatograms of non-hydrolyzed (A) and hydrolyzed (B) lignan extracted from bulked seeds of wild type CDC Bethune and M2 families 1230 and 2340, identified to carry nonsense mutations in UGT74S1. (A) lignan macromolecule profiles for the wild type CDC Bethune, M2 family 1230 predicted to have a premature stop codon at amino acid 205, and M2 family 2340, heterozygous for SNP-1859 and predicted to encode a wild type allele of UGT74S1 gene and a mutant one with a premature stop codon at amino acid 355. Note the narrower width of the lead peak in family 1230 compared to CDC Bethune as indicated by the two arrows and additional peaks on the lead peak in family 2340 as indicated by stars compared to CDC Bethune and M2 family 1230. The patterns of both M2 families also displayed additional and higher peaks (indicated by stars) in other parts of the chromatograms compared to CDC Bethune. (B) UPLC chromatograms of the hydrolyzed lignan complex from CDC Bethune, M2 families 1230 and 2340 showing peak height variations for SDG and phenolic acid glucosides encountered in the complex lignan polymers. [file Image2.JPEG]

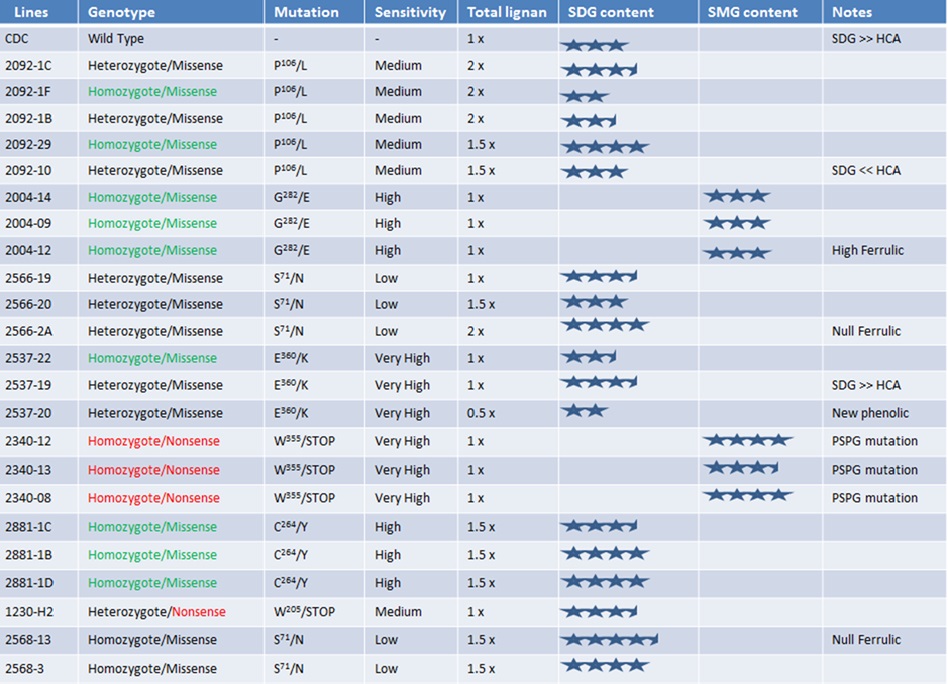

Supplement: Supplementary Figure 3 — Summary of mutational effects on lignan glucoside species production in UGT74S1 flax mutant lines. Number of stars represents the relative amount of lignan species; HCA, hydroxycinnamic acid. [file Image3.JPEG]
