# Supplementary material for: Induced Mutagenesis in UGT74S1 Gene Leads to Stable New Flax Lines with Altered Secoisolariciresinol Diglucoside (SDG) Profiles
Source: Front Plant Sci. 2017 Sep 21;8:1638. doi: 10.3389/fpls.2017.01638 (PMC5613138; doi:10.3389/fpls.2017.01638)
Supplement: Supplementary file 6 [file Table3.DOCX]

**Supplementary Table 3.** Reduced dataset of 28 M2 families selected for SNP re-validation by KASP genotyping.

| **Line #** | **Lines**  **ID #** | **Number of M2 Plants**  **genotyped*** | **M2 genotype**  **By sequencing** | **Type of Mutation** |
| --- | --- | --- | --- | --- |
| 0 | CDC | 1 | wild Homozygote | Wild type |
| 1 | 12 | 1 | Heterozygote | Missense |
| 2 | 306 | 1 | Heterozygote | Missense |
| 3 | 463 | 1 | Heterozygote | Missense |
| 4 | 756 | 1 | Heterozygote | Missense |
| 5 | 828 | 1 | Homozygote | Nonsense |
| 6 | 919 | 1 | Heterozygote | Nonsense |
| 7 | 936 | 1 | Heterozygote | Missense |
| 8 | 1230 | 1 | Homozygote | Nonsense |
| 9 | 1427 | 1 | Heterozygote | Missense |
| 10 | 1470 | 1 | Heterozygote | Missense |
| 11 | 1689 | 1 | Heterozygote | Missense |
| 12 | 1767 | 1 | Heterozygote | Nonsense |
| 13 | 1777 | 1 | Heterozygote | Nonsense |
| 14 | 1885 | 1 | Heterozygote | Nonsense |
| 15 | 2004 | 1 | Homozygote | Missense |
| 16 | 2010 | 1 | Homozygote | Missense |
| 17 | 2092 | 1 | Homozygote | Missense |
| 18 | 2340 | 1 | Heterozygote | Nonsense |
| 19 | 2517 | 1 | Heterozygote | Missense |
| 20 | 2525 | 1 | Heterozygote | Missense |
| 21 | 2526 | 1 | Heterozygote | Missense |
| 22 | 2537 | 1 | Heterozygote | Missense |
| 23 | 2566 | 1 | Homozygote | Missense |
| 24 | 2568 | 1 | Heterozygote | Missense |
| 25 | 2741 | 1 | Heterozygote | Missense |
| 26 | 2800 | 1 | Heterozygote | Missense |
| 27 | 2801 | 1 | Heterozygote | Missense |
| 28 | 2881 | 1 | Homozygote | Missense |

*Note: Different plants from the same seed lot were genotyped by sequencing and KASP
