# Supplementary material for: Induced Mutagenesis in UGT74S1 Gene Leads to Stable New Flax Lines with Altered Secoisolariciresinol Diglucoside (SDG) Profiles
Source: Front Plant Sci. 2017 Sep 21;8:1638. doi: 10.3389/fpls.2017.01638 (PMC5613138; doi:10.3389/fpls.2017.01638)
Supplement: Supplementary file 8 [file Table5.DOCX]

**Supplementary Table 5.** List and characteristics of M4 plants used in the targeted custom gene panel Ampliseq sequencing. A total of 84 M4 plants from 14 lines (1230-16, 1230-17, etc, as examples) in six families (1230, 2004, 2340, 2566, 2568, and 2881) were tested along with 9 plants from 5 CDC Bethune lines.

|  | Family ID# | Line ID# | M4 plant ID in each line | Number of M4 plants |
| --- | --- | --- | --- | --- |
| M4 | 1230 | 16 | 1A, 2A, 3A, 3B, 4A, 5A, 5B | 7 |
|  |  | 17 | 4A, 11A, 12A, 13B, 14B, 15B | 6 |
| M4 | 2004 | 9 | 1A, 1B, 2A, 4B, 11A, 11B, 13A, 15B | 8 |
|  |  | 12 | 1A, 2A, 3B, 4A, 4B, 5A, 5B | 7 |
|  |  | 14 | 1A, 1B, 3A, 5B, | 4 |
| M4 | 2340 | 8 | 1B, 2A, 2B, 3A, 3B, 4A, 4B, 5B | 8 |
|  |  | 12 | 1A, 1B, 2A, 2B, 4B, 5A, 5B | 7 |
|  |  | 13 | 2A, 3B, 5A | 3 |
| M4 | 2566 | 2A | 1B, 2A, 2B, 3B, 5A | 5 |
| M4 | 2568 | 3 | 1A, 1B, 2A, 2B, 4A | 5 |
|  |  | 13 | 1B, 2A, 2B, 3B, 4A, 4B, 5A | 7 |
| M4 | 2881 | 1B | 1B, 2B, 3A, 3B, 4A, 5A | 6 |
|  |  | 1C | 1A, 1B, 2A, 2B, 4A, 5A | 6 |
|  |  | 1D | 1A, 1B, 2A, 2B, 3A, | 5 |
| Total M4 | 6 | 14 |  | 84 |
| Wild | CDC Bethune | 1, 2, 3, 4, 5 | 1A, 1B, 2A, 2B, 3A, 3B, 4A, 5A, 5B | 9 |
